# From Endothelial Barrier Dysfunction to Circulating Biomarker: Clinical Potential of Claudin-5 in Thoracic Aortic Aneurysm and Dissection

**DOI:** 10.3390/jcm15031219

**Published:** 2026-02-04

**Authors:** Qianhui Ding, Xueyuan Yang, Zitian Duan, Haibing Li, Shuzheng Yuan, Wei Kong, Qingbian Ma, Xin Cong

**Affiliations:** 1 Department of Physiology and Pathophysiology, State Key Laboratory of Vascular Homeostasis and Remodeling, Peking University School of Basic Medical Sciences, Beijing 100191, China; dingqh@bjmu.edu.cn (Q.D.); 2216394071@bjmu.edu.cn (X.Y.); 2311210028@stu.pku.edu.cn (Z.D.); 2311110074@bjmu.edu.cn (H.L.); 2111110002@bjmu.edu.cn (S.Y.); kongw@bjmu.edu.cn (W.K.); 2 Department of Emergency, Peking University Third Hospital, Beijing 100191, China

**Keywords:** endothelial cell, biomarker, tight junction, claudin-5, thoracic aortic aneurysm and dissection

## Abstract

**Background and Objectives**: Thoracic aortic aneurysm and dissection (TAAD) is a life-threatening vascular disease with limited effective diagnostic and therapeutic strategies. Although endothelial barrier dysfunction represents an early event in TAAD pathogenesis, the role of endothelial tight junction proteins remains largely undefined. In this study, we systematically explored the function of claudin-5 (CLDN5), an endothelial-specific tight junction sealing protein, in TAAD through integrated bioinformatic, clinical, and experimental approaches. **Materials and Methods**: In the study, we combined bioinformatic analysis of the CLDN5 gene with clinical and cellular investigations. The clinical cohort included 44 patients with thoracic aortic dissection (TAAD) and 41 healthy controls. Plasma CLDN5 levels were measured by ELISA. Cellular studies involved treating human umbilical vein endothelial cells (HUVECs) with tumor necrosis factor-α (TNF-α) and performing CLDN5 knockdown, with barrier function assessed using transendothelial electrical resistance and permeability assays. **Results**: Plasma CLDN5 was significantly elevated in TAAD patients (14.20 ± 1.394 ng/mL) compared to controls (6.061 ± 0.8208 ng/mL, *p* < 0.05) and showed strong diagnostic potential with an area under the receiver operating characteristic curve (AUC) of 0.7877 (95% CI: 0.6897–0.8857). In cellular experiments, TNF-α treatment induced the release of CLDN5 fragments into the supernatant and reduced membrane CLDN5. Furthermore, CLDN5 knockdown directly impaired endothelial barrier function. **Conclusions**: Our findings identify CLDN5 as a promising circulating biomarker for TAAD diagnosis and provide new insights into TAAD pathogenesis, offering potential diagnostic strategies.

## 1. Introduction

Claudin-5 (CLDN5) is an endothelial-specific tight junction protein that serves as a critical determinant of vascular barrier integrity. This 22 kDa transmembrane protein forms the primary sealing component of endothelial tight junctions, creating a selective barrier that controls the passage of ions and small molecules while preventing the extravasation of inflammatory cells into the lesions [1]. CLDN5 loss is closely associated with inflammatory infiltration across various pathological contexts [2,3]. Its role in maintaining central nervous system homeostasis via regulation of blood–brain barrier function is well-established [4]. Beyond neurological disorders, CLDN5 dysregulation facilitates tumor metastasis by enhancing vascular permeability for cancer cell dissemination and contributes to kidney disease through the disruption of glomerular filtration barriers [5,6].

Thoracic aortic aneurysm and dissection (TAAD) represents a life-threatening cardiovascular condition characterized by progressive aortic dilation and acute dissection—a catastrophic event involving disruption of the intimal layer and separation of the aortic wall, often with fatal consequences [7]. Current understanding of TAAD pathogenesis predominantly focuses on medial degeneration and smooth muscle cell dysfunction [8]. We previously found that the disruption of endothelial barrier function is a critical early event in TAAD development, serving as both a potential predictive indicator and a promising therapeutic target [9]. Notably, CLDN5 is downregulated in the aortic tissues of the β-aminopropionitrile (BAPN)-induced TAAD mouse model [9]. However, the precise role of CLDN5 in TAAD pathogenesis remains largely unexplored.

TAAD is first suspected clinically when a patient presents with sudden-onset, severe chest pain radiating to the back syncope, or an acute pulse or blood pressure differential. Bedside transthoracic echocardiography can give the first hint of an intimal flap or pericardial effusion, but the diagnosis is confirmed via computed-tomography arteriography (CTA) that shows the dissection flap and maps the extent of the tear [10]. In stable or familial cases, magnetic resonance imaging (MRI) or trans-oesophageal echo is used to serially measure aortic diameter; dilatation ≥ 5 cm, rapid growth ≥ 0.5 cm/yr, or family history of dissection at <5 cm are accepted imaging criteria for elective diagnosis [11]. Although Lu et al. found an elevated osteoprotegerin/tumour necrosis factor-related apoptosis-inducing ligand (TRAIL) ratio, associated with increased mortality in type A acute aortic dissection, no definitive serum biomarkers are clinically available for the early diagnosis of TAAD [12]. Instead, the diagnosis of TAAD still relies heavily on imaging techniques such as CTA [13]. The absence of reliable circulating biomarkers represents a significant clinical gap, particularly for screening high-risk populations and enabling timely intervention before catastrophic aortic events occur. Intriguingly, while traditionally regarded as a transmembrane component, CLDN5 can be detected in the bloodstream, with plasma levels correlating with stroke type and location [14]. Given that TAAD initiation fundamentally involves intimal injury and that CLDN5 is a fundamental component of endothelial tight junctions, we hypothesized that endothelial damage may lead to disintegration of CLDN5 protein and release of CLDN5 soluble fragments into the circulation. Thus, a key question worth exploring is whether CLDN5 can function as a sensitive biomarker for the early detection of TAAD.

In this study, we performed integrated bioinformatic analysis of CLDN5 structure, predicting potential proteolytic cleavage sites and phosphorylation motifs that may regulate its stability and function in vascular pathology. We then used enzyme-linked immunosorbent assay (ELISA) and revealed elevated plasma CLDN5 in TAAD patients. In parallel, we examined the effect of tumor necrosis factor-α (TNF-α) on CLDN5 expression and function in human umbilical vein endothelial cells (HUVECs). Our findings suggest circulating CLDN5 as a novel and promising TAAD biomarker, while the loss of endothelial CLDN5 contributes to endothelial barrier disruption.

## 2. Materials and Methods

### 2.1. Analysis of CLDN5 Gene

The nucleotide sequence of the CLDN5 gene was downloaded from the Ensembl database (ENSG00000184113), and the JASPAR and PROMO databases were used to predict potential promoters and transcription factors binding sites (TFBS) for CLDN5 [15].

The EMBOSS_CpGplot tool [16] was employed to identify putative CpG islands using the following criteria [17]: Observed/Expected ratio > 0.60; Percent C + Percent G > 50.00; and Length > 200.

### 2.2. Analysis of CLDN5 Protein

The amino acid sequence of the CLDN5 protein was retrieved from the UniProt database (UniProtKB ID: O00501), and transmembrane topology and signal peptide prediction were jointly assessed using the Phobius database [18]. Secondary structure prediction, along with the identification of intrinsically disordered regions, was performed with PSIPRED v.3.3 and its integrated disorder predictor DISOPRED2 [19].

### 2.3. Analysis of CLDN5 Interactors

The GeneMANIA database [20] was employed to identify CLDN5 interactors, with gene descriptions retrieved from GeneCards [21]. Subsequently, functional enrichment analysis of disease-related Gene Ontology (GO) terms for the CLDN5 interactome was conducted using the ToppFun (https://toppgene.cchmc.org/enrichment.jsp, accessed on 26 November 2025) application within the ToppGene Suite [22].

### 2.4. Analysis of CLDN5 Phosphorylation and Proteolytic Cleavage Sites

To identify putative phosphorylation sites, the protein sequence were analyzed with the NetPhos 3.1 server, which assesses the likelihood of phosphorylation at serine, threonine, and tyrosine residues [23]. The potential cleavage sites within the CLDN5 protein sequence (UniProt ID: O00501) were predicted using the PeptideCutter tool. This analysis provides the query sequence with mapped proteolytic cleavage sites and a positional table of cleavage events resulting from specific proteases or chemical agents [24].

### 2.5. Measurement of Plasma Claudin-5 Level

Plasma samples from 44 patients with TAAD and 41 healthy individuals were obtained with informed consent, in collaboration with the Emergency Department of Peking University Third Hospital (M2020410, Peking University Third Hospital Medical Science Research Ethics Committee). The concentration of claudin-5 in plasma was quantified using a commercial ELISA kit (E-EL-H1630, Elabscience, Wuhan, China) according to the manufacturer’s instructions. For this kit, the manufacturer-reported lower limit of detection (LLoD) is 0.1 ng/mL, with intra- and inter-assay coefficients of variation (CVs) both <10%.

### 2.6. Cell Culture

HUVECs were cultured in endothelial cell medium (ECM, 1001, ScienCell, Carlsbad, CA, USA) supplemented with 5% fetal bovine serum (FBS) and maintained at 37 °C in a humidified 5% CO_2_ atmosphere.

### 2.7. Immunofluorescence Staining

The distribution of CLDN5 in HUVECs was visualized via immunofluorescence staining. Briefly, 48 h after TNF-α stimulation, HUVECs were fixed with 4% paraformaldehyde, permeabilized with 0.1% Triton X-100 for 5 min and blocked with 3% bovine serum albumin (BSA). Cells were then incubated overnight at 4 °C with a primary antibody against CLDN5 and incubated for 2 h at room temperature in the dark with Alexa Fluor 555-conjugated goat anti-mouse secondary antibodies (Life Technologies, San Diego, CA, USA). Nuclei were counterstained with 4′,6-diamidino-2-phenylindole (DAPI, Thermo Fisher Scientific, Rochester, NY, USA), and the stained cells were preserved in phosphate buffered saline (PBS) for subsequent confocal imaging.

### 2.8. Detection of CLDN5 in Cell Culture Supernatant

Cell culture supernatants were collected and centrifuged at 1000× *g* for 10 min at 4 °C to remove any cellular debris. The concentration of CLDN5 in cell culture supernatants was quantified using a human CLDN5 ELISA kit (CSB-EL005507HU, Cusabio, Baltimore, MD, USA) according to the manufacturer’s instructions. The manufacturer specifications for this kit report a sensitivity of 7.8 pg/mL. The intra-assay CV is <8%, and the inter-assay CV is <10%.

### 2.9. siRNA Transfection

Small interfering RNA (siRNA) against human CLDN5 and scramble siRNA were designed and synthesized by Suzhou GenePharma (Suzhou, China), and siRNA (50 nmol/L) transfection in HUVECs was applied by CALNP™ RNAi in vitro reagent (DN001, D-Nano Therapeutics, Beijing, China) according to the manufacturer’s protocol. The siRNA sequence was as follows: 5′-3′ sense: GACUACGACAAGAAGAACUTT and 5′-3′ antisense: 5′-3′ AGUUCUUCUUGUCGUAGUCTT.

### 2.10. Measurement of Transendothelial Electrical Resistance (TER)

Cells were seeded onto 0.4 μm pore-size polyester Transwell inserts (Corning) and cultured until a stable, confluent monolayer was formed. TER was measured using a volt-ohm meter (EVOM2, World Precision Instruments, Sarasota, FL, USA). Briefly, the short and long electrode tips were placed in the upper and lower chambers, respectively, and the measured resistance (Ω) was multiplied by the surface area of the membrane (cm^2^) to calculate the TER value (Ω·cm^2^).

### 2.11. Paracellular Permeability Assay

Paracellular permeability to macromolecules was assessed using 4 kDa FITC-labeled dextran (1 mg/mL; FD4, Sigma-Aldrich, St. Louis, MO, USA). After TER measurement, the cell monolayers were rinsed and equilibrated with transport buffer. FITC-dextran solution was added to the upper chamber, while fresh buffer was placed in the lower chamber. The plate was incubated for 3 h at 37 °C, after which samples were collected from the lower chamber. The fluorescence intensity (Ex/Em: 490/520 nm) was measured using a microplate reader.

### 2.12. Statistical Analysis

All the experiments were performed in triplicate (unless otherwise specified) in at least three independent experiments, and all the statistical analyses were performed using GraphPad Prism 10.1 software (GraphPad Software, San Diego, CA, USA). The values are presented as the means ± standard errors (SEMs) for normally distributed data. For comparisons of normally distributed data between two groups, we first assessed the homogeneity of variances using an F-test or Levene’s test. If variances were similar, Student’s t-test was applied; if not, Welch’s t-test was used. The normal distribution of the data was first evaluated using the Shapiro-Wilks test. For comparisons of normally distributed data between two groups, we applied Student’s *t* test for data with similar variances. In addition, for comparisons of more than two groups, the Brown–Forsythe test was used to assess similar variances, followed by ordinary ANOVA or Welch’s ANOVA test when similar variances were or were not assumed, respectively. The Mann–Whitney U test for 2 groups or the Kruskal–Wallis test with Dunn’s multiple comparisons test for >2 groups was applied to data that were not normally distributed. Specifically, Dunnett’s multiple comparisons test was used to compare each group with a control group, while Tukey’s multiple comparisons test was used to compare each group with every other group. A *p* value of <0.05 was considered to indicate statistically significant. Representative images were chosen to most accurately represent the group mean/average across all the available data.

## 3. Results

### 3.1. Circulating CLDN5 Serves as a Potential Biomarker for TAAD

**Figure 1:**
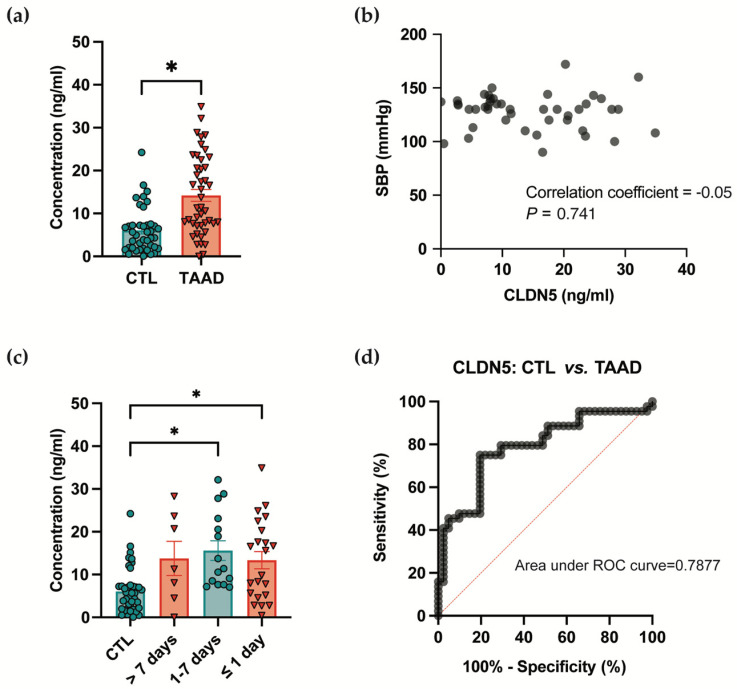
Plasma claudin-5 (CLDN5) is elevated in thoracic aortic aneurysm and dissection (TAAD) patients. (**a**) Plasma CLDN5 concentrations in TAAD patients (N = 44, red) and healthy controls (CTL, N = 41, green). * *p* < 0.05 using Welch’s *t* test; (**b**) Correlation between plasma CLDN5 levels and systolic blood pressure (SBP) in TAAD patients (N = 44); (**c**) CLDN5 levels relative to time before symptom onsets (CTL, N = 41; time ≤ 1-day, N = 22; time > 1-day and ≤7-day, N = 15; time > 7-day, N = 7). * *p* < 0.05 using the Kruskal–Wallis test with Dunn’s multiple comparisons test; (**d**) ROC curve analysis evaluating the diagnostic performance of CLDN5 for TAAD.

Considering that CLDN5 loss has been discovered in aortic tissues of TAAD, we explored whether CLDN5 could be cleaved and released into the blood circulation. A case–control study comprising 44 TAAD patients and 41 healthy controls was conducted. As summarized in Table 1, the two groups showed no significant differences in gender (*p* = 0.7722) or age (*p* = 0.6309), which are risk factors associated with TAAD [3]. Other basic clinical characteristics are presented in Table 1. ELISA measurements revealed a marked increase in plasma CLDN5 levels in TAAD patients (14.20 ± 1.394 ng/mL) compared to healthy controls (6.061 ± 0.8208 ng/mL; Figure 1a). We further found that the CLDN5 levels were not correlated with systolic blood pressure using Spearman’s or Pearson’s correlation tests (Figure 1b), suggesting that elevated plasma CLDN5 may represent an independent risk indicator for TAAD. We also investigated whether CLDN5 levels were associated with the time interval of sample collection prior to acute TAAD symptom onset. Among the 44 TAAD patients, 22 samples were obtained within 1 day, 15 between 1 and 7 days, and 7 after 7 days before symptom onset (Figure 1c). Compared to the control group (CTL: 6.061 ± 0.8208 ng/mL), plasma CLDN5 levels were higher in >7-day group (13.77 ± 3.995 ng/mL). CLDN5 levels continuing to rise until their peak during the 1 to 7 days immediately preceding symptom onset (15.62 ± 2.289 ng/mL). The levels remained highly elevated even within the final 24 h before the acute event (13.36 ± 2.006 ng/mL). These findings suggest that circulating CLDN5 levels rise dynamically in the period preceding an acute TAAD, with the most prominent increase occurring within one week before symptom onset. The receiver operating characteristic (ROC) curve analysis showed that the circulating CLDN5 level exhibited a diagnostic value of 0.7877 (95% CI: 0.6897–0.8857; *p* < 0.0001) for discriminating TAAD patients from healthy controls (Figure 1d). In both healthy individuals and TAAD patients, changes in plasma CLDN5 levels are not associated with hypertension (Figure S1a,b). No mortality was documented in the TAAD cohort throughout the study period; accordingly, the potential association between plasma CLDN5 levels and fatal outcomes could not be evaluated.

Even after adjusting for systolic blood pressure, diastolic blood pressure, age, and gender, its association remains highly significant (Figure S1c, odds ratio: 1.082, *p* < 0.0001). This means that between two individuals with identical blood pressure, age, and gender, the one with a higher level of this biomarker has a significantly increased risk of TAAD. Its discriminatory value is independent of all these traditional factors.

### 3.2. Loss of CLDN5 Leads to Disrupted Endothelial Barrier Function

**Figure 2:**
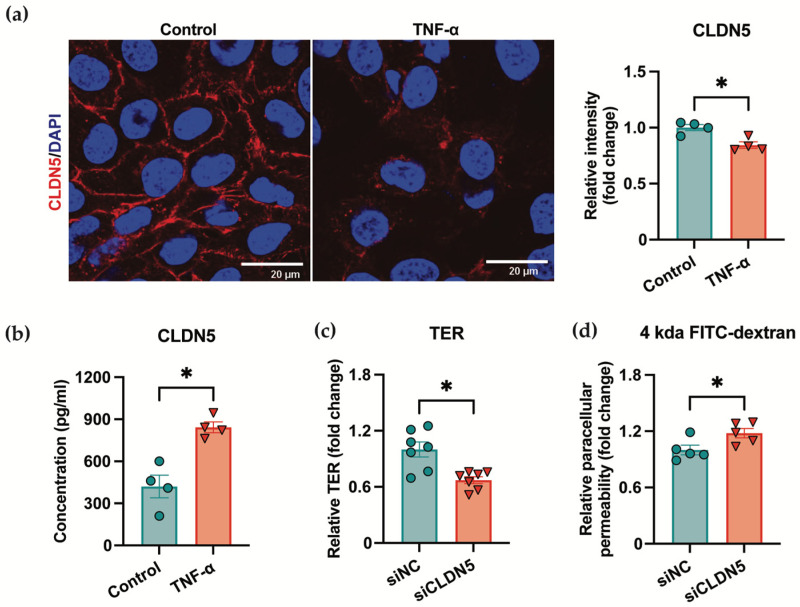
Tumor necrosis factor-α (TNF-α) disrupts endothelial barrier integrity through claudin-5 (CLDN5) release and functional impairment: (**a**) Immunofluorescence images showing CLDN5 expression (red) in human umbilical vein endothelial cells (HUVECs) with or without TNF-α stimulation. Nuclei were counterstained with DAPI (blue). Data are presented as the mean ± SEM and analyzed by using an unpaired two-tailed Student’s *t* test. N = 4 per group. Scale bar = 20 μm. * *p* < 0.05; (**b**) CLDN5 concentrations in HUVECs culture supernatants with or without TNF-α stimulation quantified via ELISA. Data are presented as the mean ± SEM and analyzed by using an unpaired two-tailed Student’s *t* test. N = 4 per group. * *p* < 0.05; (**c**) Transendothelial electrical resistance (TER) of HUVECs under control conditions and after CDLN5 knockdown. Data are presented as the mean ± SEM and analyzed by using an unpaired two-tailed Student’s *t* test. N = 7 per group. * *p* < 0.05. (**d**) Paracellular permeability assay assessed using 4 kDa FITC-dextran in HUVECs under control conditions and after CDLN5 knockdown. Data are presented as the mean ± SEM and analyzed by using an unpaired two-tailed Student’s *t* test. N = 5 per group. * *p* < 0.05.

To determine whether alteration in CLDN5 localization underlies the increase in plasma CLDN5 under pathological conditions, we treated HUVECs with TNF-α, a key inflammatory trigger in TAAD. Following 48 h TNF-α treatment, CLDN5 expression in the plasma membrane was dramatically reduced, while soluble CLDN5 levels in the culture supernatant significantly increased, as measured via ELISA (Figure 2a,b). The substantial reduction in membrane CLDN5 observed via immunofluorescence may reflect both its proteolytic release and internalization/degradation [25], whereas ELISA specifically quantifies the shed extracellular fragment.

To further assess the functional role of CLDN5, we performed siRNA-mediated knockdown in HUVECs. CLDN5 knockdown in HUVECs, with an efficiency determined by validating the reduction in mRNA level by qRT-PCR and Western blot. As shown in Supplementary Figure S2, transfection with siCLDN5 significantly reduced CLDN5 expression in HUVECs compared to the control siRNA group. CLDN5 knockdown resulted in a significant decrease in TER and an increase in paracellular flux of a fluorescent probe (Figure 2c,d), indicating impaired endothelial barrier integrity. Collectively, these findings suggest that inflammation-induced loss of membranous CLDN5 compromises endothelial barrier function, and that the concomitant release of CLDN5 into the extracellular compartment may underlie its potential as a circulating biomarker for TAAD.

### 3.3. CLDN5 Interaction Network Implicates Its Role in Vascular Function and Disease

**Figure 3:**
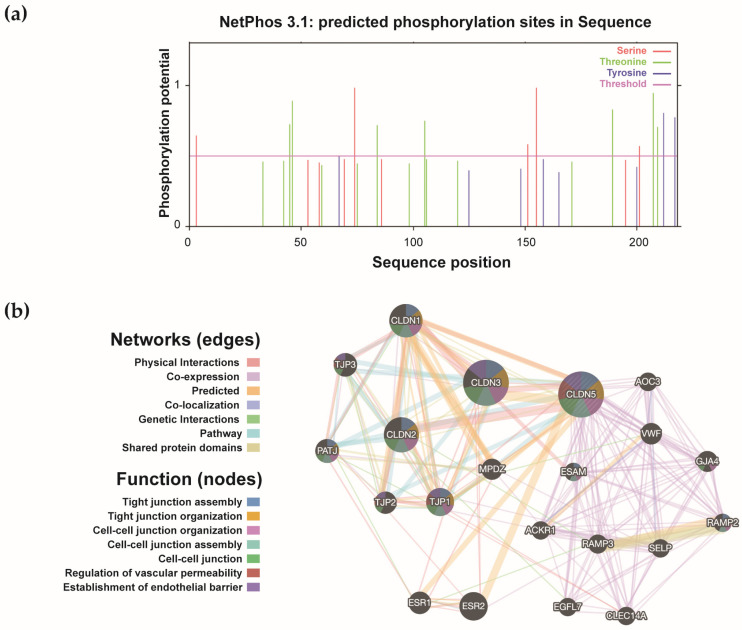
Claudin-5 (CLDN5) phosphorylation site and interaction network analysis: (**a**) Predicted high-confidence (score > 0.5) phosphorylation sites for serine, threonine, and tyrosine residues; (**b**) Protein–protein interaction (PPI) network depicts functional association via physical interactions, co-expression, and pathway enrichment. Nodes represent proteins, edges represent predicted functional associations.

Next, to determine the mechanism underlying the cleavage and release of CLDN5, we identified CLDN5 phosphorylation sites including Ser155, Ser74, Thr207, Thr46, and Tyr212/217 via NetPhos 3.1 in silico predictions (Table S1 and Figure 3a). These sites were distributed across cytoplasmic and extracellular domains, with specific kinases such as protein kinase C (PKC), PKA, and ataxia-telangiectasia mutated (ATM) predicted to target distinct residues, and sites may also regulate CLDN5 stability and susceptibility to proteolysis. The inflammatory signaling in TAAD might promote CLDN5 release via coordinated phosphorylation and proteolytic events. Based on the proteolytic analysis using PeptideCutter, the CLDN5 protein sequence contained cleavage sites targeted by a range of enzymatic and chemical reagents (Table S2). Notably, several predicted cleavage sites are located in the first extracellular loop (e.g., after residues Arg30 and Lys65), which, if cleaved, could potentially release a soluble fragment containing a major portion of the extracellular domain into circulation. Highly specific proteases such as trypsin and Arg-C proteinase cleaved at 11 and 6 sites, respectively, while broad-specificity enzymes like proteinase K recognized over 130 cleavage positions. It should be noted that the prediction for broad-specificity proteases (e.g., proteinase K) has limited biological interpretability, whereas predictions for proteases with defined roles in vascular remodeling are of greater mechanistic interest. These modification patterns provide possible mechanistic insights into how CLDN5 might be released into circulation through phosphorylation-induced shedding and/or proteolytic cleavage.

Moreover, we explored the function of CLDN5 and bioinformatic profiling defined a CLDN5-centric interaction network comprising 21 genes (Figure 3b and Table 2). Analysis of these genes revealed highly concentrated functions, primarily involved in cell junction assembly, tight junction formation and maintenance, and regulation of vascular endothelial barrier function (Table 3). Functional enrichment analysis of diseases was strongly enriched in vascular pathologies, including hypertensive disease, coronary artery disease, myocardial infarction, and transient cerebral ischemia (Table S3). These results suggested that CLDN5 may have a broad involvement in vascular homeostasis and diseases. These results, along with established research, suggest that CLDN5 plays a broad role in vascular homeostasis, and its dysregulation contributes to disease pathogenesis. For instance, recent research has demonstrated that SARS-CoV-2 disrupts the respiratory endothelial barrier primarily by downregulating CLDN5 expression, and therapeutic restoration of CLDN5 can rescue this barrier dysfunction, underscoring its pivotal and targetable role in vascular integrity during infection [26].

### 3.4. Integrated Analysis Shows the Translational and Post-Translational Modification of CLDN5

**Figure 4:**
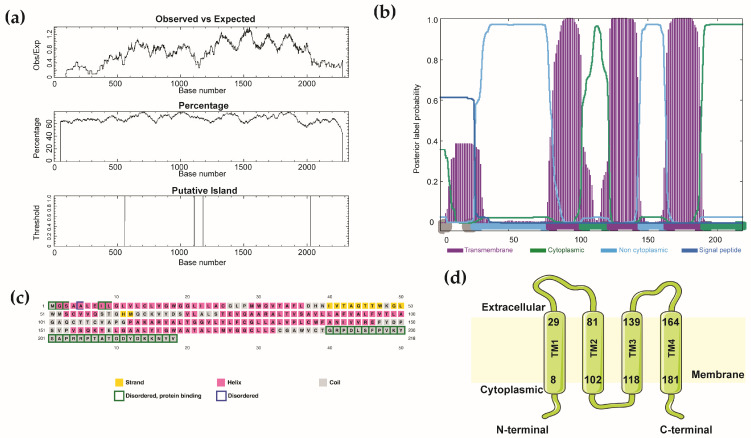
Structural profiling of claudin-5 (CLDN5) reveals a four-pass transmembrane protein with defined regulatory features: (**a**) CpG island analysis of the CLDN5 gene identifying two islands (positions 560–1109, length 550 bp; and 118–2028, length 848 bp) across the analyzed sequence (1–2332 bp); (**b**) Predicted membrane topology of CLDN5 generated using the Phobius database. Transmembrane domains are shown in purple, intracellular regions in green, and extracellular regions in blue; (**c**) Secondary structure and disorder propensity predictions; (**d**) Schematic illustrating the predicted domain architecture of CLDN5 protein.

Then we analyzed the potential transcription factors that regulated claudin-5 expression through promoter analysis using Promo and JASPAR. Multiple transcription factor binding sites (TFBS) were identified and distributed across both DNA strands at various positions (Table 4). Key predicted transcription factors include estrogen receptor α (ERα), paired box 5 (PAX5), activating protein 2α (AP2α), and CCAAT/enhancer-binding protein α (C/EBPα), with ERα showing the highest prediction score, suggesting that CLDN5 expression may be regulated by diverse signaling pathways involved in hormone response, immune regulation, and cellular stress. Analysis of human CLDN5 gene identified two CpG islands (Figure 4a), supporting that the possibility that epigenetic regulatory mechanisms may influence its expression. Structural analysis using Phobius and PSIPRED computationally confirmed that CLDN5 adopted a four-transmembrane topology with cytoplasmic N- and C-terminal tails (Figure 4b–d). These promoters and structural in silico analyses establish that CLDN5 expression is potentially governed by a complex network involving specific transcription factors and epigenetic mechanisms, underpinning its roles in vascular biology and disease.

## 4. Discussion

**Figure 5:**
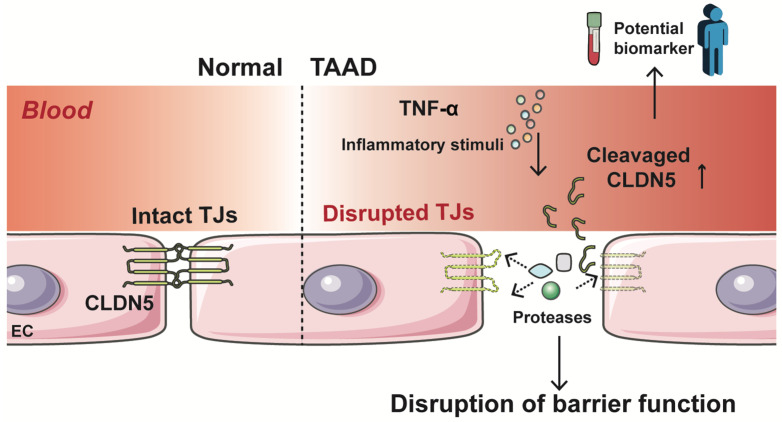
Schematic summarizing the role of CLDN5 in thoracic aortic aneurysm and dissection (TAAD). Under physiological conditions, endothelial cells (ECs) form intact tight junctions (TJs), with CLDN5 maintaining endothelial barrier integrity. In TAAD, inflammatory stimuli (e.g., TNF-α) induce the proteolytic cleavage of CLDN5 by proteases, disrupting TJs and releasing CLDN5 fragments into circulation as potential biomarkers.

The intact endothelial barrier is pivotal for vascular homeostasis. In this study, through an integrated approach combining bioinformatic analyses, clinical sample validation, and in vitro functional experiments, the role of endothelial-specific tight junction transmembrane protein CLDN5 in TAAD was explored. We found distinctive regulatory features and an interaction network of CLDN5 and revealed a significant elevation of circulating CLDN5 levels in TAAD patients (a proposed model in Figure 5). Our findings suggest that loss of CLDN5 in endothelial cells contributes to TAAD pathogenesis, and more importantly, CLDN5 may serve as a potential biomarker for TAAD.

A major finding of this study is the potential of circulating CLDN5 as a TAAD biomarker. Although the loss of endothelial tight junction contents and impairment of endothelial barrier function have been identified to be an early event in the occurrence and progression of TAAD [9], whether the tight junction proteins could be cleaved and released into circulation during TAAD was still unknown. Furthermore, the clinical role of a circulating biomarker like CLDN5 may differ across the disease spectrum. In chronic thoracic aortic aneurysm, its levels might reflect the burden of ongoing endothelial dysfunction, aiding in risk stratification. In contrast, a sharp, acute rise could signal the transition to dissection, providing a potential window for emergency intervention. Previous studies detected CLDN5, occludin, and ZO-1 in plasma, and found the levels of CLDN5 and occludin to be positively associated with disease progression in elderly patients with Alzheimer’s disease [27]. CLDN5 and junctional adhesion molecule-A (JAM-A), two major tight junction proteins important for endothelial barrier integrity, have been reported as substrates for ADAM17 (a disintegrin and metalloproteinase-17), a membrane-bound enzyme that regulates the bioavailability of multiple transmembrane proteins through proteolytic processing [28]. Beta-site amyloid precursor protein cleaving enzyme 1 (BACE1) establishes its pathological role in cleaving occludin and disrupting the cerebral microvascular barrier [29]. These studies indicated that the release of tight junction protein fragments into the bloodstream may be an indicator of vascular pathologies, potentially through proteolytic cleavage [30]. Here, our bioinformatic analysis identified several potential proteolytic cleavage sites within the CLDN5 protein sequence. The in silico cleavage site analysis presented here is exploratory. While it highlights potential sites for proteases like matrix metalloproteinases (MMPs), the predictions require experimental validation. Furthermore, the analysis did not specifically screen for or prioritize proteases with established upregulation in aortic aneurysms and dissections (e.g., MMP2, MMP9, and ADAM10/17). Future work should focus on experimentally validating cleavage by these specific enzymes in disease-relevant models. Critically, the level of circulating CLDN5 levels in the plasma of TAAD patients was significantly higher than that of controls. Notably, this elevation was dynamic before the acute event, peaking most prominently within one week before symptom onset. Moreover, TNF-α stimulation increased the level of CLDN5 in the culture supernatants of HUVECs, again indicating that CLDN5 fragments can be released from endothelial cells and are detectable in extracellular fluid. Thus, our novel findings provided evidence showing that elevated circulating CLDN5 may serve as an efficient indicator of vascular endothelial injury, making it a valuable biomarker for the diagnosis of acute TAAD.

Another finding of this study is the identification of CLDN5 absence in TAAD pathogenesis. The genetic deletion of CLDN5 in mice resulted in a selective leak of the blood–brain barrier to small molecules, while the overall vascular structure remained intact, identifying CLDN5 as the primary sealing protein of the endothelial tight junctions [31]. The link between claudin-5 loss and barrier failure is a fundamental mechanism in vascular disease, as evidenced by research showing that its specific down-regulation is sufficient to induce blood–brain barrier breakdown under inflammatory central nervous system (CNS) conditions [32]. Here, we established an in vitro endothelial cell injury model that mimics the vascular microenvironment in TAAD [33]. Our results showed that TNF-α stimulation induced the disappearance of CLDN5 in the cell membranes, accompanied by an increase in CLDN5 levels in supernatants. Furthermore, the lack of CLDN5 led to impaired endothelial barrier function, manifested through decreased TER and increased permeability. These results strongly suggest that CLDN5 is not merely a passive marker of endothelial injury but plays an active role in maintaining vascular barrier function. The inflammation-induced downregulation and altered spatial distribution of CLDN5 may represent a crucial mechanism underlying vascular barrier disruption in the development and progression of TAAD.

However, a critical consideration for the potential clinical translation of CLDN5 as a TAAD biomarker is its specificity. While our study demonstrates a significant elevation in TAAD patients compared to healthy controls, we did not measure CLDN5 levels in patients with other acute cardiovascular events, such as myocardial infarction, pulmonary embolism, or stroke. It is plausible that conditions involving significant endothelial injury or systemic inflammation could also lead to elevated circulating CLDN5 levels. For instance, acute MI involves coronary artery endothelial disruption, and severe inflammatory states could potentially cause widespread endothelial activation. Therefore, the diagnostic specificity of CLDN5 for distinguishing TAAD from other acute conditions, particularly in an emergency department setting where such differential diagnoses are common, remains unknown and requires dedicated investigation. Future studies must include well-defined control groups comprising patients with these acute vascular and inflammatory conditions to establish the specificity and positive predictive value of CLDN5 for TAAD. A biomarker that cannot reliably differentiate aortic dissection from acute myocardial infarction would have limited standalone utility in triage but could potentially be valuable as part of a multi-marker panel or in conjunction with clinical assessment and imaging.

Despite these meaningful findings, this study has several limitations. First, the clinical sample size, while reasonable for this initial investigation, is relatively small. Multi-center, large-scale prospective studies are required to robustly validate the diagnostic accuracy, establish reliable cut-off values, and assess the clinical utility of CLDN5. The proposed mechanism of CLDN5 release via proteolytic cleavage remains hypothetical and requires direct experimental validation. Second, while HUVECs serve as a suitable model for studying basic endothelial cell functions and are of venous origin, they may not fully recapitulate the phenotypic, functional, and shear-stress response profiles of arterial endothelial cells, particularly those of the thoracic aorta. Therefore, our cellular findings, while informative, should be interpreted within the context of this model limitation. Future studies employing primary aortic endothelial cells (e.g., HAECs) or more complex arterial-specific models are warranted to confirm the inflammatory response and CLDN5 release dynamics in a context more directly relevant to TAAD pathophysiology. Furthermore, the precise molecular mechanisms governing the release of CLDN5 from endothelial junctions, including the specific proteases involved, require further in-depth investigation. Third, the absence of mortality events in our cohort during the study period precluded an assessment of whether CLDN5 levels correlate with fatal outcomes.

## 5. Conclusions

In this study, we systematically reveals the important predictive role of CLDN5 in TAAD, providing substantial evidence for its utility as a novel biomarker and experimental support for its functional role in the pathogenesis of TAAD. These findings offer new insight into the diagnosis of TAAD and lay a theoretical foundation for developing protective therapeutic strategies targeting the endothelial barrier.

## Figures and Tables

**Table 1:** Clinical and biochemical characteristics of TAAD patients and healthy participants included in the study.

CLDN5 Study	TAAD	CTL	*p* Value
n	44	41	-
Gender (male)	36 (81.8%)	36 (87.8%)	0.7722
Age (year)	48.27 ± 13.46	47.12 ± 7.48	0.6309
Hypertension	32 (72.7%)	2 (4.9%)	<0.05 *
CLDN5 (ng/mL)	14.20 ± 1.394	6.061 ± 0.8208	<0.05 **

TAAD thoracic aortic aneurysm and dissection, CTL control; * *p* < 0.05 by Fisher’s exact test, ** *p* < 0.05 by Mann–Whitney test.

**Table 2:** Key genes in the network.

Gene Symbol	Gene Name
ACKR1	Atypical chemokine receptor 1
AOC3	Amine oxidase copper containing 3
CLDN1	Claudin-1
CLDN2	Claudin 2
CLDN3	Claudin 3
CLEC14A	C-type lectin domain containing 14A
EGFL7	EGF like domain multiple 7
ESAM	Endothelial cell adhesion molecule
ESR1	Estrogen receptor 1
ESR2	Estrogen receptor 2
GJA4	Gap junction protein alpha 4
MPDZ	Multiple PDZ domain crumbs cell polarity complex component
PATJ	PATJ crumbs cell polarity complex component
RAMP2	Receptor activity modifying protein 2
RAMP3	Receptor activity modifying protein 3
SELP	Selectin P
TJP1	Tight junction protein 1
TJP2	Tight junction protein 2
TJP3	Tight junction protein 3
VWF	Von Willebrand factor

**Table 3:** Key genes enriched Gene Ontology biological processes.

Function	Genes in Network	Genes in Genome
Apical junction assembly	8	68
Tight junction assembly	8	69
Tight junction organization	8	73
Tight junction	8	79
Apical junction complex	8	83
Cell–cell junction organization	9	169
Cell–cell junction assembly	8	104
Cell–cell junction	9	272
Regulation of vascular permeability	5	25
Establishment of endothelial barrier	5	30
Tissue homeostasis	7	163
Endothelial cell development	5	55
Endothelial cell differentiation	5	81
Endothelium development	5	103
Epithelial cell development	5	114
Bicellular tight junction assembly	3	19
Vascular process in circulatory system	5	185
Cellular component maintenance	3	40
Cell–cell adhesion via plasma-membrane adhesion molecules	4	171

**Table 4:** Prediction of promoters and transcription factor binding sites (TFBS) by Promo and JASPAR.

Matrix ID	Name	Score	Relative Score	Sequence	Start	End	Strand	Predicted Sequence
MA0258.2	ERα	15.4695	0.93413	CLDN5	1213	1227	−	AGGTCACTGTGGCTT
MA0014.2	PAX5	14.7741	0.900168	CLDN5	482	500	−	CTGGGCAACAGAGTGAGAC
MA0003.2	AP2α	14.7519	0.925774	CLDN5	1892	1906	−	CCCTGCCCCAGGGTA
MA0466.1	C/EBPα	12.2369	0.948208	CLDN5	86	96	−	GGTTTCACCAT
MA0007.2	AR	12.1988	0.906431	CLDN5	1693	1707	−	AAGCACAGTGTGGCC
MA0113.1	GR	11.2149	0.852941	CLDN5	1032	1049	−	GAGACCAGCCTGTCCAAC
MA0051.1	IRF2	11.194	0.800563	CLDN5	775	792	−	CTAAAGAGAAAGTCTTAA
MA0670.1	NFI	10.8633	0.959406	CLDN5	34	43	−	TTTGCCAATC
MA0095.2	YY1	10.3135	0.874514	CLDN5	82	93	+	CAACATGGTGAA
MA1146.1	RXRα	9.45291	0.811961	CLDN5	505	519	+	GAGTGCAGTGACACA
MA0850.1	FOXP3	9.35863	0.951146	CLDN5	1717	1723	+	GCAAACA
MA0106.2	p53	8.75483	0.820605	CLDN5	1118	1132	+	GCTTGGCCTGGCTTG
MA0518.1	STAT4	6.94723	0.843777	CLDN5	386	399	−	CTTGTTGGAAACAG
MA0108.2	TFIID	6.9413	0.825268	CLDN5	112	126	+	ATTTAAAAAACAAAA
MA0108.2	TFII-I	6.9413	0.825268	CLDN5	112	126	+	ATTTAAAAAACAAAA

Sequence: identifier of the DNA sequence used for prediction analysis; start: starting position of the predicted binding site in the sequence; end: ending position of the predicted binding site in the sequence; strand: DNA strand orientation of the predicted binding site (forward “+” or reverse “−“); predicted sequence: specific DNA sequence fragment predicted to be the transcription factor binding site.

## Data Availability

Data is unavailable due to privacy and ethical restrictions.

## Supplementary Materials

The following supporting information can be downloaded at: https://www.mdpi.com/article/10.3390/jcm15031219/s1, Figure S1: Analysis of plasma claudin-5 (CLDN5) levels in relation to hypertension status; Figure S2: Validation of claudin-5 (CLDN5) knockdown efficiency in human umbilical vein endothelial cells (HUVECs); Table S1: Prediction of Protein Phosphorylation Sites by NetPhos 3.1 (Score > 0.5); Table S2: Potential cleavage sites in the protein sequence were predicted using the PeptideCutter tool, which maps the locations of enzymatic and chemical proteolysis; Table S3: Functional enrichment analysis of diseases associated with CLDN5 interactome.

## Abbreviations

The following abbreviations are used in this manuscript:

TAAD	Thoracic aortic aneurysm and dissection
CLDN5	Claudin-5
HUVEC	Human umbilical vein endothelial cells
ELISA	Enzyme-linked immunosorbent assay
FBS	Fetal bovine serum
TFBS	Transcription factor binding sites
