# Supplementary material for: From Endothelial Barrier Dysfunction to Circulating Biomarker: Clinical Potential of Claudin-5 in Thoracic Aortic Aneurysm and Dissection
Source: J Clin Med. 2026 Feb 4;15(3):1219. doi: 10.3390/jcm15031219 (PMC12898148; doi:10.3390/jcm15031219)
Supplement: Supplementary file 1 [file jcm-15-01219-s001.zip › jcm-4040873-supplementary.pdf]

Supplemental materials

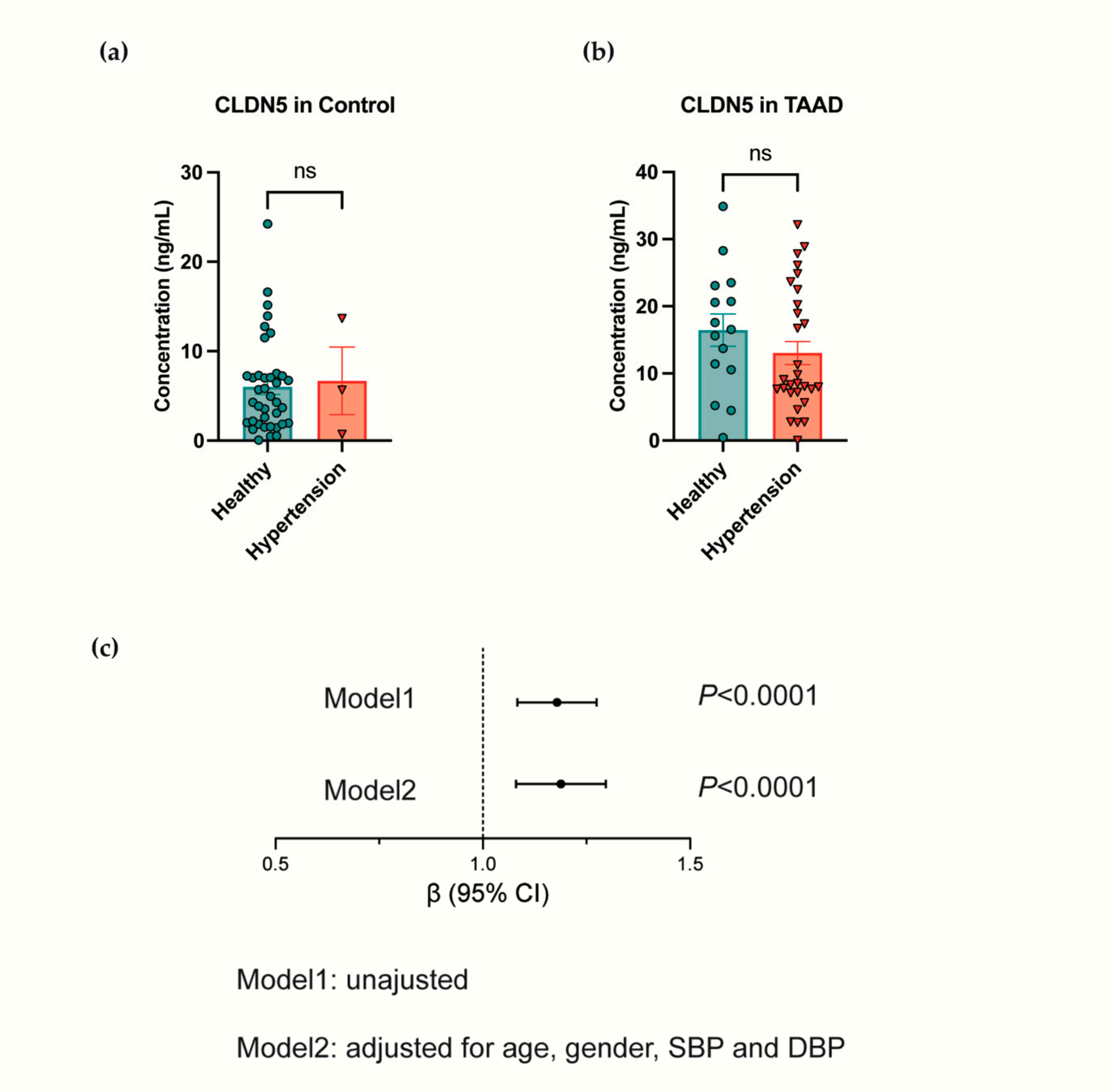

Figure S1. Analysis of plasma claudin-5 (CLDN5) levels in relation to hypertension status. (a) Plasma CLDN5 concentrations in healthy controls, stratified by hypertension status (normotensive vs. hypertensive). Data are presented as mean ± SEM. Statistical analysis was performed using Mann-Whitney U test. N=3-38; NS, not significant. (b) Plasma CLDN5 concentrations in TAAD patients, stratified by hypertension status (normotensive vs. hypertensive). Data are presented as mean ± SEM. Statistical analysis was performed using Mann-Whitney U test. N=3-38; NS, not significant. (c) Forest plots showing the adjusted odds ratio (95% confidence interval) for plasma cldn5 levels associated with TAAD using the cohort data. Binary logistic regression was adjusted for age, gender, systolic blood pressure (SBP), and diastolic blood pressure (DBP).

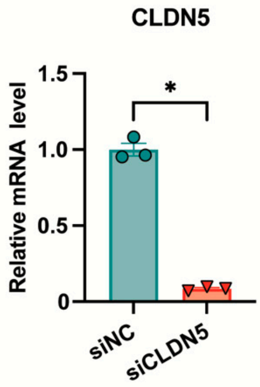

Figure S2. Validation of claudin-5 (CLDN5) knockdown efficiency in human umbilical vein endothelial cells (HUVECs). (A) Relative CLDN5 mRNA levels in HUVECs transfected with control siRNA (siNC) or CLDN5-specific siRNA (siCLDN5), measured by qRT-PCR (n=3).

Table S1. Prediction of protein phosphorylation sites by NetPhos 3.1 (Score > 0.5)

Position	x	Context	Score	Kinase	Answer
155	S	SVPVSQKYE	0.983	unsp	YES
74	S	VLALSTEVQ	0.978	unsp	YES
207	T	PRRPTATGD	0.944	unsp	YES
46	T	TAQTTWKGL	0.890	unsp	YES
189	T	AWVCTGRPD	0.824	unsp	YES
212	Y	ATGDYDKKN	0.802	unsp	YES
217	Y	DKKNYV	0.772	unsp	YES
105	T	GAQCTTCVA	0.747	unsp	YES
45	T	VTAQTTWKG	0.720	unsp	YES
84	T	ARALTVSAV	0.716	unsp	YES
209	T	RPTATGDYD	0.703	PKC	YES
45	T	VTAQTTWKG	0.683	PKC	YES
207	T	PRRPTATGD	0.658	PKG	YES
3	S	MGSAALE	0.640	PKA	YES
155	S	SVPVSQKYE	0.605	ATM	YES
151	S	FYDPSVPVS	0.577	PKC	YES

201	S	PVKYSAPRR	0.569	PKC	YES
84	T	ARALTVSAV	0.562	PKC	YES
207	T	PRRPTATGD	0.542	PKA	YES
105	T	GAQCTTCVA	0.531	PKC	YES
74	S	VLALSTEVQ	0.522	cdc2	YES

Context: the amino acid sequence fragment encompassing the phosphorylation site of interest; score: The prediction confidence score (ranging from 0-1). A score > 0.5 is generally considered a positive prediction; Kinase: the protein kinase predicted to poten-tially catalyze phosphorylation at this site. "unsp" indicates that no specific kinase was predicted; answer: "YES" indicates a posi-tive result (i.e., score > 0.5). PKA, protein kinase A; PKC, protein kinase C; ATM, ataxia-telangiectasia mutated.

Table S2. Potential cleavage sites in the protein sequence were predicted using the PeptideCutter tool

Name of Enzyme	No. of Cleavages	Positions of Cleavage Sites
Arg-C proteinase	6	81 116 145 191 204 205
Asp-N endopeptidase	6	36 67 148 192 210 212
Asp-N endopeptidase + N-terminal Glu	10	6 36 67 75 145 148 158 192 210 212
BNPS-Skatole	7	18 30 47 51 138 168 186
CNBr	5	1 29 52 62 175
Chymotrypsin-high specificity (C-term to [FYW], not before P)	20	18 30 35 47 67 92 96 125 127 138 139 147 148 158 165 168 186 200 212 217
Chymotrypsin-low specificity (C-term to [FYWML], not before P)	56	1 6 9 11 13 15 18 21 23 29 30 35 36 38 47 50 52 62 67 71 73 83 89 90 92 95 96 99 119 124 125 126 127 130 131 133 136 138 139 147 148 158 160 164 165 168 173 174 175 180 181 186 194 200 212 217
Clostripain	6	81 116 145 191 204 205
Formic acid	6	37 68 149 193 211 213
Glutamyl endopeptidase	4	7 76 146 159
Iodosobenzoic acid	7	18 30 47 51 138 168 186
LysC	7	48 65 114 157 199 214 215
LysN	7	47 64 113 156 198 213 214
NTCB (2-nitro-5-thiocyanobenzoic acid)	12	13 24 53 63 103 106 127 136 178 181 182 187
Pepsin (pH1.3)	59	5 6 8 9 10 11 12 13 14 15 20 21 22 23 27 34 35 36 49 70 71 72 73 82 88 89 90 91 92 94 95 96 98 99 119 123 124 125 126 127 129 130 131 132 135 138 139 146 160 163 164 172 173 174 179 180 181 194 196

Pepsin (pH>2)	78	5 6 8 9 10 11 12 13 14 15 17 18 20 21 22 23 27 30 34 35 36 46 47 49 51 66 70 71 72 73 82 88 89 90 91 92 94 95 96 98 99 119 123 124 125 126 127 129 130 131 132 135 137 138 139 146 157 158 160 163 164 165 167 168 172 173 174 179 180 181 185 186 194 196 199 200 211 212
Proline-endopeptidase	2	192 206
Proteinase K	130	4 5 6 7 8 9 11 12 13 15 16 18 21 22 23 24 27 30 32 33 34 35 36 40 41 42 43 45 46 47 50 51 55 56 59 66 67 70 71 72 73 75 76 77 79 80 82 83 84 85 87 88 89 90 91 92 93 94 95 96 97 98 99 100 102 105 106 108 109 113 115 117 118 119 120 123 124 125 126 127 130 131 132 133 134 136 138 139 140 142 143 144 146 147 148 152 154 158 159 160 162 163 164 165 166 168 169 170 171 172 173 174 176 180 181 185 186 187 189 194 196 198 200 202 207 208 209 212 217 218
Staphylococcal peptidase I	4	7 76 146 159
Thermolysin	89	3 4 5 8 10 11 12 14 15 20 21 22 23 28 31 33 34 35 39 40 42 49 51 54 55 61 65 69 70 71 72 78 79 81 82 84 86 87 88 89 90 91 92 93 94 95 96 98 99 101 107 112 114 116 117 118 122 123 125 126 129 130 131 132 135 138 139 141 142 143 153 161 162 163 165 168 169 171 172 173 174 175 179 180 184 186 197 207 217
Trypsin	11	48 65 81 114 116 145 157 199 204 214 215

Table S3. Functional enrichment analysis of diseases associated with claudin-5 (CLDN5) interactome

ID	Name	Source	p-value	q-value Bonferroni	q-value FDR B&H	q-value FDR B&Y	Hit Count in Query List	Hit Count in Genome
DOID:8634	prostate carcinoma in situ (implicated via orthology)	AllianceGenome	4.824E-07	3.763E-04	3.763E-04	2.723E-03	2	2
DOID:8283	peritonitis (implicated via orthology)	AllianceGenome	1.447E-06	1.128E-03	5.642E-04	4.083E-03	2	3
C0020538	hypertensive disease	DisGeNET Curated	9.149E-06	7.137E-03	2.379E-03	1.722E-02	4	190
DOID:224	transient cerebral ischemia (implicated via orthology)	AllianceGenome	1.259E-05	9.818E-03	2.454E-03	1.776E-02	3	64
C0282612	prostatic intraepithelial neoplasias	DisGeNET Curated	2.643E-05	2.062E-02	3.645E-03	2.638E-02	2	11
DOID:13976	peptic esophagitis (biomarker via orthology)	AllianceGenome	3.17E-05	2.473E-02	3.645E-03	2.638E-02	2	12
DOID:5844	myocardial infarction (is implicated in)	AllianceGenome	4.663E-05	3.637E-02	3.645E-03	2.638E-02	3	99
DOID:3393	coronary artery disease (is implicated in)	AllianceGenome	4.805E-05	3.748E-02	3.645E-03	2.638E-02	3	100
C0033578	prostatic neoplasms	DisGeNET Curated	6.017E-05	4.693E-02	3.645E-03	2.638E-02	5	616
C0376358	malignant neoplasm of prostate	DisGeNET Curated	6.017E-05	4.693E-02	3.645E-03	2.638E-02	5	616

The analysis was performed using ToppFun (ToppGene Suite). The top 10 significantly enriched terms are listed. Abbreviations: FDR, False Discovery Rate; B&H, Benjamini-Hochberg; B&Y, Benjamini-Yekutieli.
